# Ethical and Legal Aspects of Informed Consent and Assent in Paediatric Dentistry: A Cross-Sectional Study

**DOI:** 10.3390/children12121711

**Published:** 2025-12-18

**Authors:** Maria Josefa Ferro de Farisato-Touceda, Laura Marqués-Martínez, Esther García-Miralles, Juan Ignacio Aura-Tormos, Clara Guinot Barona

**Affiliations:** 1 Dentistry Department, Faculty of Medicine and Health Sciences, Catholic University of Valencia San Vicente Mártir, 46001 Valencia, Spain; mj.ferro@ucv.es (M.J.F.d.F.-T.); clara.guinot@ucv.es (C.G.B.); 2 Faculty of Medicine and Dentistry, University of Valencia, 46010 Valencia, Spain; m.esther.garcia@uv.es (E.G.-M.); juan.aura@uv.es (J.I.A.-T.)

**Keywords:** informed consent, assent, paediatric dentistry, healthcare decision-making

## Abstract

**Highlights:**

**What are the main findings?**

- Children received information on diagnosis, treatment and benefits consistently, but key elements such as risks, alternatives and the right to withdraw consent were often omitted.
- Child schooling level—not caregiver education—was the strongest independent predictor of receiving more complete information within the consent process.

**What are the implications of the main findings?**

- Standardised consent and assent protocols are needed to ensure that all paediatric patients, regardless of age or schooling, receive comprehensive and ethically adequate information.
- Enhancing bioethical and communication training for dental students may improve the consistency and quality of informed consent practices in paediatric dentistry.

**Abstract:**

Background: Informed consent and assent are fundamental ethical and legal requirements in paediatric healthcare, yet their application in paediatric dentistry is complex and underexplored in clinical practice. Objective: This study aimed to analyse the implementation of informed consent and assent processes in paediatric dental care within a Spanish population, identifying key characteristics and factors that influence communication, understanding, and decision-making. Methods: An observational, descriptive, cross-sectional study was conducted in Spanish Paediatric Dentistry Clinics (January–June 2023). Participants included 520 child-caregiver pairs and 52 dental students. Data were collected via a semi-structured observational protocol and interviews, assessing information provided, decision-making conditions, and influencing factors. Statistical analysis was performed using SPSS v23.0, employing Chi-square, Cochran’s Q, and Kendall’s W tests. Results: The information most frequently provided was the nature of the dental problem (92%), treatment details (88%), and benefits (85%). Information on risks (64%), alternatives (37%), and the right to withdraw consent (41%) was less consistently communicated. After multivariable adjustment, child schooling remained independently associated with the disclosure of risks and alternatives (*p* < 0.01), whereas caregiver education showed no independent effect. Kendall’s concordance coefficient showed moderate agreement (W = 0.62, 95% CI: 0.54–0.69, *p* < 0.01) among operators, caregivers, and patients, which decreased in adolescents aged 16–18 years (W = 0.41, 95% CI: 0.28–0.55, *p* = 0.07). Conclusions: The processes of informed consent and assent in paediatric dentistry are more strongly linked to the child’s cognitive maturity and schooling than to parental education. While communication of treatment benefits is adequate, critical aspects like risks and alternatives are often overlooked. The findings underscore the need for standardized protocols and enhanced bioethical training to ensure consistent, ethical, and participatory practices that respect the progressive autonomy of minors.

## 1. Introduction

The principles of informed consent and assent in minors are grounded in respect for personal autonomy, a central concept in moral philosophy and modern bioethics [1]. In the biomedical field, informed consent is formalized through major international documents such as the International Ethical Guidelines for Biomedical Research Involving Human Subjects [2] and the Universal Declaration of Human Rights [3]. In Spain, this principle has been translated into an extensive legal framework that protects patient rights and the autonomy of decision-making. These include the Spanish Constitution [4], General Health Law 14/1986 [5], Law 41/2002 on Patient Autonomy [6], Law 1/2003 on Patients’ Rights and Information in the Valencian Community [7], Law 26/2011 on adaptation to the Convention on the Rights of Persons with Disabilities [8], Law 15/2015 on Voluntary Jurisdiction [9], Law 26/2015 on the protection of children and adolescents [10], and the Spanish Code of Ethics and Dental Deontology [11].

According to Law 41/2002, informed consent is defined as “the free, voluntary and conscious conformity of a patient, expressed in full possession of his faculties after receiving adequate information, so that an action affecting his health may take place” (Art. 3) [6]. The concept of assent, meanwhile, is reflected in Law 15/2015, which establishes the obligation of healthcare professionals to listen to minors’ opinions in all matters affecting their health [9]. Spanish law sets 16 years as the age of majority in health care [6]; however, the participation of younger minors through assent remains ethically and practically relevant when accompanied by parental or guardian consent, balancing legal protection with the gradual development of autonomy [12,13].

In paediatric dentistry, obtaining valid consent and assent presents specific challenges. Dental treatments often generate anxiety, involve complex technical explanations, and require adapting information to a child’s cognitive and emotional level. Ensuring that minors participate in decisions about their own health encourages moral development, responsibility, and adherence to preventive and therapeutic measures—extending beyond the traditional risk–benefit framework.

These statements are supported by previous research showing that dental anxiety, communication complexity, and the need to tailor information to children’s developmental stages are recurrent challenges in paediatric dental encounters [14]. Furthermore, studies in paediatrics and child psychology indicate that involving minors in health decisions promotes moral development, autonomy, adherence, and cooperative behaviour [15]. However, despite clear ethical and legal guidelines, empirical evidence on how informed consent and assent are operationalised in real paediatric dental practice remains scarce.

Despite the existence of clear ethical and legal guidelines, few empirical studies have examined how informed consent and assent are actually applied in paediatric dental practice in Spain or other European contexts. Most available studies have focused primarily on caregivers’ perspectives or on professionals’ self-reported practices, often through interviews or surveys, with limited direct observation of real clinical interactions. Moreover, few investigations have simultaneously analysed the views of children, caregivers, and student operators within the same encounter, which restricts understanding of how consent and assent are operationalized in everyday paediatric dental settings. A better understanding of this process is crucial for strengthening communication between dentists, patients, and caregivers, improving ethical clinical practice, and designing educational tools that promote respect for autonomy in child healthcare.

Therefore, this study aimed to analyse the process of informed consent and assent in paediatric dental care within a Spanish population, identifying its main characteristics and the factors influencing communication, understanding, and decision-making among students, patients, and caregivers.

This study was guided by principles of Child- and Family-Centred Care (CFCC) and the concept of progressive autonomy, which emphasise the child’s evolving capacity, shared decision-making, and the need to adapt information to developmental stages [16,17].

## 2. Materials and Methods

### 2.1. Study Design and Setting

An observational, descriptive, and analytical cross-sectional study was designed and conducted in accordance with the STROBE (Strengthening the Reporting of Observational Studies in Epidemiology) guidelines for reporting observational research.

The study was carried out between January and June 2023 in the Paediatric Dentistry Clinics of Spain, which provide both educational and clinical care for children and adolescents. These clinics serve a heterogeneous population of patients from diverse socioeconomic and educational backgrounds, allowing for the assessment of different levels of understanding and participation in the consent process.

The main objective was to analyse how the processes of informed consent and assent were implemented in paediatric dental care, exploring communication, comprehension, and decision-making among children, caregivers, and dental students. The aim was not to audit student performance but to document and analyse communication and consent practices across children, caregivers, and student operators.

### 2.2. Participants

The study population included paediatric dental patients aged 2 to 18 years, their parents or legal caregivers, and undergraduate dental students performing clinical rotations. Participants were selected through consecutive sampling of all eligible patients attending their first visit or routine check-up during the study period.

Children and adolescents accompanied by a caregiver able to provide consent and attending for preventive or conservative dental treatment were eligible. Patients or caregivers who declined participation, emergency visits where information could not be fully provided, or cases involving severe communication difficulties that prevented effective interviews were excluded. In total, 520 child–caregiver pairs and 52 dental students participated in the study.

For the purposes of this study, adolescents aged 16 years and older were considered to have partial decision-making autonomy in accordance with Spanish Law 41/2002 on Patient Autonomy, which establishes 16 as the legal age of majority for health-related decisions. Assent was obtained from minors under 16, and informed consent from caregivers was required in all cases. According to Spanish Law 41/2002, adolescents aged 16–18 provided informed consent. Children aged 6–15 were invited to provide assent, which was evaluated through their ability to restate information, answer simple understanding checks, and demonstrate behavioural engagement. Children under 6 received age-appropriate oral explanations, and observational assent was considered based on comfort, cooperation, and non-verbal cues. Comprehension was assessed by the trained researcher conducting the interview, using developmentally adapted questions and behavioural indicators.

In all cases, caregivers received and signed a written informed consent document. Children were provided with an oral, age-appropriate explanation of the procedure, adapted to their level of understanding. Both the caregiver and the child were given the opportunity to read or listen to the relevant information before the observational and interview protocol was administered.

Caregivers were asked to read the written consent prior to signing, while children received a verbal explanation to ensure their comprehension could be assessed during the research protocol.

### 2.3. Variables and Data Collection

Data were collected using a semi-structured observational protocol designed by experts in bioethics and paediatric dentistry. The instrument assessed: (1) information provided to the patient and caregiver (nature of the disease, treatment details, risks, benefits, confidentiality, and alternatives); (2) conditions for decision-making (understanding, freedom, competence); (3) influencing factors (education, persuasion, manipulation, coercion); and (4) time allocated for decision-making.

The instrument was developed through expert consultation, a pilot test, and a reliability assessment using Cronbach’s alpha (α = 0.84), indicating good internal consistency. Data collection involved direct participatory systematic observation, recorded on standardized forms, and complemented by semi-structured interviews. Interviews were carried out in two stages: first with the child and caregiver together immediately after the appointment, and subsequently with the student-operator at the end of clinical practice.

The observation tool used Likert-type scales, dichotomous items, and categorical descriptors. Coding rules and scoring procedures were standardised between observers. The tool was developed through expert consultation (paediatric dentists and bioethicists), pilot testing with five clinical encounters, and iterative revision to refine clarity, relevance, and developmental appropriateness.

For analytic purposes, patients were categorised according to educational stage (preschool, primary, secondary, and high school), which reflects developmental differences more accurately than chronological age alone. This classification was used in both descriptive and multivariable analyses.

Here, ‘participatory’ refers to the observer’s physical presence during the clinical encounter, allowing direct observation of natural communication without interference or participation in the procedure.

Interviews were conducted immediately after the appointment to ensure accuracy of recall. They lasted approximately 6–10 min and were held in a private area within the clinic. Interviews were not audio-recorded to avoid discomfort. Questions were adapted to children’s developmental stages, using simplified language or visual supports when needed. No compensation was offered to avoid undue influence.

Three adapted versions of the tool were used: one for observation, one for the child/caregiver interview, and one for the student interview. All versions shared core constructs but items were phrased differently according to the respondent.

### 2.4. Bias Control

To minimize bias, a data masking technique was applied so that dental students were unaware of which of their patients had been included in the study. Interviewers were trained to use a standardized script to reduce interviewer bias, and observations were recorded independently by two researchers, with discrepancies resolved by consensus.

### 2.5. Statistical Analysis

Data were analysed using SPSS v23.0 (IBM Corp., Armonk, NY, USA) and R version 4.3.0 (R Foundation for Statistical Computing, Vienna, Austria).

Categorical variables were summarized as counts and percentages, and continuous variables as the mean ± standard deviation (SD).

Group comparisons were performed using Chi-square (χ^2^) tests for independence, and the Cochran’s Q test was applied to assess within-subject differences in the relative emphasis placed on different information items (e.g., benefits vs. risks vs. alternatives).

Kendall’s coefficient of concordance (W) was used to evaluate the degree of agreement among operator, caregiver, and patient, with 95% confidence intervals (CI) estimated by percentile bootstrap with 1000 replicates.

To identify independent factors associated with the disclosure of (a) treatment risks and (b) therapeutic alternatives, two multivariable binary logistic regression models were fitted.

Covariates were pre-specified based on theoretical relevance and their bivariate associations with the outcomes, including the child’s age (treated as a continuous variable), sex (male or female), schooling level (primary, secondary, or high school), caregiver’s educational level (primary, secondary, or university), and the category of dental procedure (preventive, operative, or surgical). To account for potential intra-clinic correlation, clinic was included as a fixed effect, and robust standard errors were clustered by clinic.

Adjusted odds ratios (OR) with 95% confidence intervals (CI) were reported, and all tests were two-sided with a significance level of α = 0.05. The achieved sample size ensured a statistical power greater than 80% to detect an odds ratio of at least 1.5 for the main associations, assuming a two-tailed α = 0.05.

### 2.6. Ethical Considerations

The study followed the principles of the Declaration of Helsinki, the Universal Declaration of Human Rights, the Convention on the Rights of the Child, and the International Ethical Guidelines for Biomedical Research in Human Subjects (CIOMS/WHO), as well as relevant Spanish legislation on patient autonomy, healthcare rights, and data protection [2,3,4,5,6,7,8,9,10,11]. Ethical approval was obtained from the Research and Ethics Committee for Human Studies in Spain (Ref. No. UCV-2023-017). Written informed consent was obtained from all parents or caregivers, and assent was sought from minors according to their level of understanding. Confidentiality and anonymity were guaranteed in compliance with the Spanish Organic Law 3/2018 on the Protection of Personal Data and Guarantee of Digital Rights, which incorporates the principles of the European General Data Protection Regulation (GDPR). All clinical records, data, and audiovisual materials were used exclusively for academic and research purposes, ensuring full respect for participants’ privacy and rights.

To minimise power imbalance, only one observer was present during each appointment, the researcher was introduced as a neutral figure, and children or caregivers could request the observer to leave at any time.

Participation was voluntary at all times. Withdrawal after consent was allowed and respected, and was operationalised through verbal refusal, discomfort, or unwillingness to continue.

The reporting of this study aligns with recommendations from child-centred research checklists within the EQUATOR Network, ensuring attention to communication adaptation, child involvement, and minimisation of power imbalance.

### 2.7. Participant Characteristics

A total of 520 child–caregiver pairs and 52 dental students participated in the study. The mean age of the paediatric patients was 8.9 ± 3.6 years (range 2–18 years), with a balanced gender distribution (51% female, 49% male). Most caregivers were mothers (73%), followed by fathers (24%) and other legal guardians (3%).

Regarding educational level, 58% of caregivers had completed upper secondary education, 26% held a university degree, and 16% had only primary education. Among the dental students, 70% were in their fourth year and 30% in their fifth year of the degree programme. Table 1 presents the sociodemographic characteristics of the study population.

## 3. Results

### 3.1. Information Provided in the Consent Process

Analysis of the information disclosed during the consent process revealed that the aspects most frequently communicated by students were the nature of the disease or dental problem (92%), the details and objectives of the proposed treatment (88%), and the expected benefits (85%).

Information regarding the possible risks and complications was provided in 64% of cases, mainly focused on common side effects rather than rare or severe outcomes.

**Figure 1:**
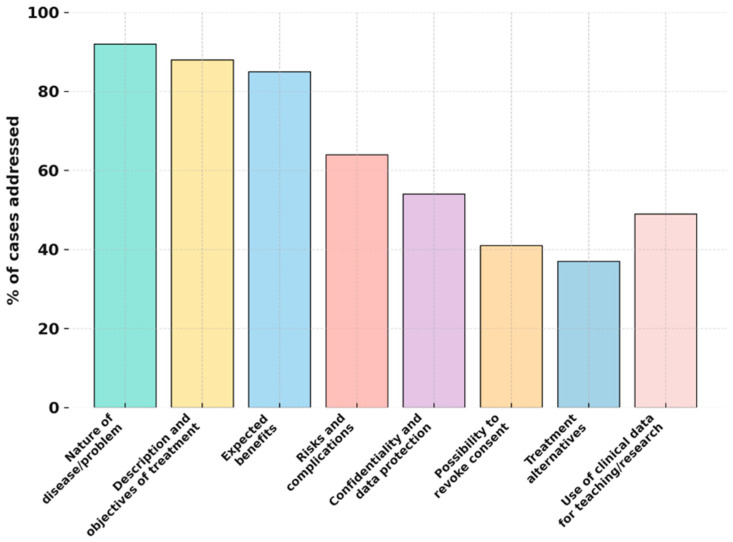
Frequency of information items provided during the consent process.

Only 41% of participants reported receiving an explanation about the possibility of revoking consent, and 37% recalled being informed about treatment alternatives. Aspects such as confidentiality guarantees (54%) and the use of clinical data for academic purposes (49%) were mentioned less frequently. Figure 1 summarises the proportion of cases in which each type of information was addressed.

### 3.2. Associations Between Consent Type and Patient or Caregiver Variables

Statistical analysis demonstrated a significant association between the type of consent (assent vs. informed consent) and the patient’s educational level (χ^2^ = 19.82, *p* < 0.001), indicating that understanding and participation increased with schooling. This evaluation was conducted across the full age range (2–18 years). For younger children with limited verbal communication, participation consisted of observational assent, consistent with ethical guidelines. Interpretation of associations therefore considered developmental differences in communication abilities.

In contrast, no significant relationship was found between consent type and the caregiver’s educational level (χ^2^ = 3.45, *p* = 0.178), suggesting that parental instruction did not substantially influence the child’s involvement in decision-making.

The Cochran’s Q test confirmed differences in the proportional emphasis of information provided: operators tended to focus more on treatment explanation and benefits, while rarely addressing risks or confidentiality (*p* < 0.01). Table 2 summarises the main associations between consent type and sociodemographic or communication variables.

### 3.3. Multivariable Analysis of Associated Factors

After adjustment, higher child schooling remained independently associated with the disclosure of treatment risks (OR = 2.38, 95% CI: 1.34–4.23, *p* = 0.003) and with the disclosure of therapeutic alternatives (OR = 1.91, 95% CI: 1.08–3.38, *p* = 0.026).

Caregiver educational level showed no independent association after controlling for other variables (*p* = 0.214).

Procedure category was also related to the likelihood of discussing risks, with operative procedures showing higher disclosure rates than preventive ones (OR = 1.87, 95% CI: 1.02–3.44, *p* = 0.041).

Child age and sex were not significant predictors in the adjusted models (both *p* > 0.10).

### 3.4. Agreement Between Operator, Caregiver, and Patient

**Figure 2:**
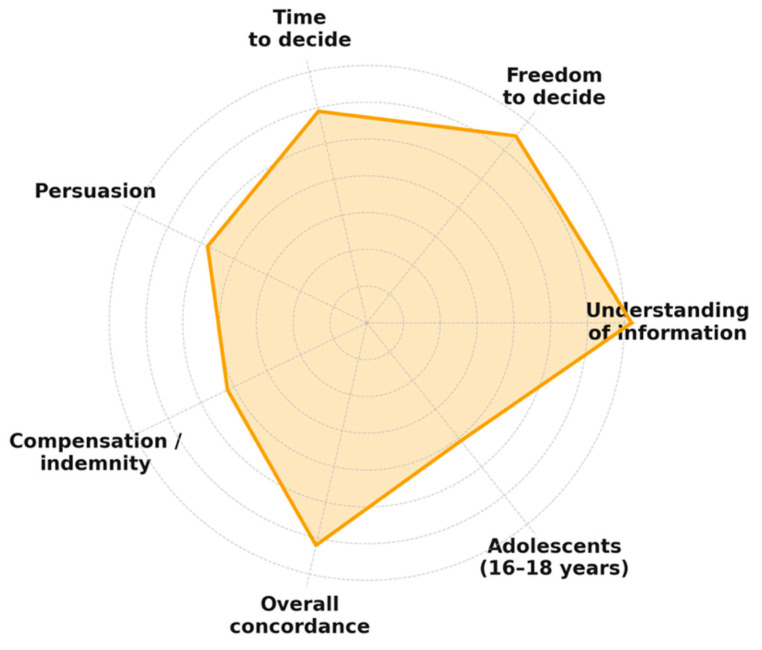
Agreement (Kendall’s W) between operator, caregiver, and patient in key consent variables.

Kendall’s coefficient of concordance indicated a moderate overall agreement among the operator, caregiver, and patient regarding the information provided (W = 0.62, 95% CI: 0.54–0.69, *p* < 0.01). The highest levels of agreement were observed in variables related to understanding and freedom to decide, while lower concordance appeared in items such as compensation and persuasion, where responses tended to polarize. In adolescents aged 16–18 years, concordance decreased (W = 0.41, 95% CI: 0.28–0.55, *p* = 0.07), reflecting greater variability between adolescents’ self-perception of autonomy and caregiver evaluation. Figure 2 displays item-level W values with 95% bootstrap confidence intervals.

Significant associations were also found between the type of consent and variables related to freedom and competence to decide, while understanding of information showed a weaker association under assent and a stronger dependency under informed consent.

## 4. Discussion

The results of this study indicate that informed consent and assent in paediatric dentistry are strongly influenced by the child’s cognitive development and schooling level. Spanish law requires that healthcare professionals listen to minors’ opinions on matters affecting their health (Ley 26/2015) [10], while Law 41/2002 establishes the age of 16 as the threshold for full health-related decision-making autonomy [6]. Accordingly, this study considered assent applicable up to 15 years and informed consent from the age of 16 onward. These criteria align with Waligora et al. [13], who advocate individualized consent models adapted to developmental stages. Younger children showed greater dependence on caregivers for decision-making, whereas adolescents demonstrated increasing comprehension and reasoning ability. Our findings align with classical theories of moral and cognitive development [18,19,20], which establish that abstract reasoning and hypothetical thinking emerge progressively during adolescence.

These findings reinforce the need for developmentally sensitive communication strategies that integrate both written and personalised oral explanations, ensuring that children of all ages meaningfully participate in decisions related to their dental care.

Although children under 5–6 years of age may not fully understand dental explanations or reliably express preferences, their inclusion in the study was necessary to capture the full developmental spectrum routinely encountered in paediatric dentistry. In accordance with Spanish ethical and legal frameworks, clinicians are expected to adapt communication to the child’s level of maturity and to promote participation through age-appropriate assent, even when the child’s role is primarily receptive. Including preschool children therefore allowed us to analyse how consent-related communication is approached from the earliest stages of development and to identify disparities in the completeness of information provided. In this regard, our findings showed that chronological age itself was not a significant predictor of receiving explanations about risks, complications, or therapeutic alternatives, whereas schooling level was. Schooling appears to reflect functional cognitive maturity, verbal comprehension, and interactional readiness more accurately than age alone, which may explain why clinicians tend to adjust the depth of their explanations based on perceived understanding rather than biological age. This distinction highlights the importance of developmentally sensitive communication strategies that ensure all paediatric patients—regardless of age or schooling—receive ethically adequate and comprehensive information.

From a clinical perspective, the observed odds ratio of 2.38 for schooling level indicates that children with higher educational attainment were more than twice as likely to receive information about potential treatment risks compared with those in primary education. This finding highlights a nontrivial communication gap: as children progress cognitively and verbally, clinicians appear to adapt their explanations, but younger patients remain at risk of receiving simplified—or incomplete—information. Such disparity suggests that information delivery may be unconsciously tailored to perceived understanding rather than to ethical obligations of disclosure. Strengthening communication strategies and using age-appropriate educational materials could help ensure that all children, regardless of schooling level, receive consistent and complete explanations of treatment risks and alternatives.

From a practical standpoint, these findings suggest that a well-structured written informed consent should always be complemented by a personalised oral explanation adapted to the child’s developmental stage. Such oral clarification may function as an essential annex to the basic legal document, ensuring that minors truly understand the treatment process.

In contrast, Baines [21] argues that assent may create confusion, suggesting that only competence should determine the validity of consent. Our findings support a middle-ground approach: recognising assent as an ethical and educational step toward autonomy, without equating it to legal consent. This perspective is supported by Boceta et al. [22], who emphasize assessing the capacity of the mature minor on a case-by-case basis.

Regarding communication, children and caregivers reported that treatment explanations and benefits were the most clearly presented, while risks, complications, and alternatives were less frequently discussed. Similar deficiencies have been noted in other studies, such as Coyne et al. [23], who highlight the insufficient adaptation of information to the minor’s language and level of understanding. Our data also showed that most minors perceived the information as understandable, even if they did not feel fully entitled to decide—reflecting a persistent gap between comprehension and autonomy. This aligns with the observations of Ruiz J. [24], who highlighted the broader challenges in the situation of minors within the healthcare context, where their autonomy is often limited despite their understanding.

The obligation to adapt communication to the child’s level of maturity and to include their perspective in clinical decision-making is consistent with Esquerda et al. [25] and Espejo et al. [26], who recommend assessing minors’ competence proportionally to the risk–benefit balance of each intervention, as proposed by Drane’s sliding scale [27]. This model, which defines three levels of capacity according to the complexity and consequences of the decision, is particularly relevant for dental care, where most procedures involve minimal or moderate risk.

Persuasion appeared necessary in certain cases involving young or behaviourally uncooperative patients. As Broggi [28] points out, persuasion may be ethically acceptable when used to promote the child’s best interest, provided the argument is truthful and non-coercive. Coercion and manipulation, however, were not observed in our study.

Finally, the time available to make health decisions was perceived as adequate in most cases, though younger children often lacked the chronological and cognitive capacity to manage time-related concepts. This observation is consistent with Esquerda et al. [25], who emphasised that decision-making time should be proportional to the gravity and potential consequences of treatment, requiring both maturity and parental involvement.

Overall, the findings underscore the need to strengthen structured protocols for informed consent and assent in paediatric dentistry, ensuring consistency, clarity, and ethical compliance across professionals. Such protocols should promote the child’s progressive autonomy while maintaining the essential triadic relationship between child, caregiver, and dentist.

The moderate concordance observed (W = 0.62) suggests a reasonable but incomplete alignment between operators, caregivers, and children in how information is perceived and understood. The decrease in W among older adolescents supports the notion of increasing autonomy and differentiated perspectives during late adolescence, consistent with previous studies on shared decision-making.

This study’s main strengths include a large, multicentric sample, the standardized observation protocol, and the triangulation of perspectives from children, caregivers, and student operators, providing a comprehensive view of the consent and assent process. The use of Cochran’s Q and Kendall’s W with bootstrap confidence intervals adds statistical robustness and supports the internal consistency of the findings.

However, some limitations should be acknowledged. The cross-sectional design precludes causal inference, and despite masking procedures, observer and social desirability biases may have occurred. Because the dental students were aware that aspects of communication and consent might be observed, a Hawthorne effect cannot be ruled out; their behaviour during the study may have reflected enhanced diligence or ethical awareness compared with routine practice. Consequently, the frequency and quality of information disclosure observed here could represent an optimistic estimation of what occurs under usual clinical conditions. In addition, although multivariable adjustment was performed, residual confounding may persist, and the academic setting limits generalizability to other paediatric dental environments.

Additionally, the subjective nature of assessing communication behaviours may influence the completeness and reliability of the observations, despite efforts to minimise bias.

## 5. Conclusions

Informed consent and assent in paediatric dentistry are closely linked to the child’s cognitive maturity and schooling, rather than the caregiver’s educational level. Communication barriers remain significant in younger children and in those with intellectual disabilities, highlighting the importance of adapting information to each child’s developmental stage. Although information about treatment and expected benefits is generally well provided, aspects such as risks, alternatives, and the right to withdraw consent are often underexplained, indicating a need for more comprehensive and balanced communication. The consent process in paediatric dental practice should be family-centred, fostering collaboration between the child, caregivers, and dentist, while recognising the progressive autonomy of minors as required by ethical and legal frameworks. Our findings suggest that informed consent is implemented reasonably well in many cases, although important aspects such as risks, alternatives, and the possibility of withdrawing consent remain insufficiently addressed. Establishing a standardised protocol for informed consent and assent would ensure greater consistency, clarity, and ethical rigour in clinical practice. Furthermore, integrating bioethical training on informed consent and assent into dental education could strengthen students’ communication skills and promote a more participatory approach to child and adolescent oral healthcare.

## Figures and Tables

**Table 1:** Sociodemographic characteristics of participants.

Variable	Category
Patients (*n* = 520)	8.9 ± 3.6 Male: 255 (49.0%)/Female: 265 (51.0%)
Age (years), mean ± SD	
Gender	
Caregivers	Mother: 379 (72.9%)/Father: 125 (24.0%)/Other: 16 (3.1%)
Relationship to child	
Educational level	Primary: 83 (16.0%)/Secondary: 302 (58.1%)/University: 135 (26.0%)
Dental students (*n* = 52)	4th year: 36 (69.2%)/5th year: 16 (30.8%)
Year of training	

**Table 2:** Statistical associations between consent type and participant variables *.

Variable	Statistical Test	χ^2^/Q Value	*p*-Value	Association
Patient’s schooling	χ^2^	19.82	<0.001	Significant
Caregiver’s education	χ^2^	3.45	0.178	Not significant
Emphasis on treatment info	Q	22.61	<0.01	Significant
Emphasis on risks/confidentiality	Q	18.34	<0.01	Significant

* Chi-square tests for independence and Cochran’s Q tests used to compare proportions of information provided.

## Data Availability

Data are not publicly available due to ethical and legal restrictions. The dataset contains sensitive information from minors and their caregivers obtained during clinical encounters, and cannot be shared openly in accordance with Spanish data protection regulations (LOPD-GDD) and the GDPR. De-identified data may be made available from the corresponding author upon reasonable request and subject to approval by the Research and Ethics Committee.

## Abbreviations

The following abbreviations are used in this manuscript:

AAP	American Academy of Pediatrics
CIOMS	Council for International Organizations of Medical Sciences
GDPR	General Data Protection Regulation
IC	Informed Consent
IA	Informed Assent
SD	Standard Deviation
SPSS	Statistical Package for the Social Sciences
STROBE	Strengthening the Reporting of Observational Studies in Epidemiology
W	Kendall’s Coefficient of Concordance
χ^2^	Chi-square test
Q	Cochran’s Q test
*p*	Probability value
CI	Confidence Interval
UCV	Universidad Católica de Valencia (used for the Ethics Committee reference)
